# Epigallocatechin Gallate-Loaded Gelatin-*g*-Poly(*N*-Isopropylacrylamide) as a New Ophthalmic Pharmaceutical Formulation for Topical Use in the Treatment of Dry Eye Syndrome

**DOI:** 10.1038/s41598-017-09913-8

**Published:** 2017-08-24

**Authors:** Li-Jyuan Luo, Jui-Yang Lai

**Affiliations:** 1 Department of Chemical and Materials Engineering, Chang Gung University, Taoyuan, 33302 Taiwan, ROC; 2 Institute of Biochemical and Biomedical Engineering, Chang Gung University, Taoyuan, 33302 Taiwan, ROC; 3 Department of Ophthalmology, Chang Gung Memorial Hospital, Taoyuan, 33305 Taiwan, ROC; 4 Department of Materials Engineering, Ming Chi University of Technology, New Taipei City, 24301 Taiwan, ROC

## Abstract

Given that biodegradable *in situ* gelling delivery systems may have potential applications in the design of ophthalmic pharmaceutical formulations, this study, for the first time, aims to develop gelatin-*g*-poly(*N*-isopropylacrylamide) (GN) carriers for topical epigallocatechin gallate (EGCG) administration in the treatment of dry eye disease (DED). By temperature triggered sol-gel phase transition of copolymers, EGCG-loaded GN was prepared at 32 °C and characterized by FTIR, NMR, and HPLC analyses. Results of WST-1 and live/dead assays showed that GN materials have good compatibility with corneal epithelial cells. Gradual biodegradation of delivery carriers allowed sustained release of EGCG without drug toxicity. Anti-inflammatory and antioxidant activity studies also indicated effective therapeutic drug levels at each time point within 3 days of release. In a rabbit dry eye model, corneal epithelial defects was ameliorated by treatment with single-dose administration of EGCG-containing GN. Furthermore, drug molecules released from carrier materials could prevent further tear evaporation and loss of mucin-secreting goblet cells in diseased animals. Our findings suggest that GN carrier is responsible for enhanced pharmacological efficacy of topically instilled EGCG, thereby demonstrating the benefits of using biodegradable *in situ* gelling delivery system to overcome the drawbacks of limited dry eye relief associated with eye drop dosage form.

## Introduction

Pharmacological treatment of inflammatory dry eye disease (DED) has attracted much attention due to the prevalence and morbidity of this public health problem. Topical ocular application of corticosteroid (i.e., anti-inflammatory medication) is a commonly used treatment modality to effectively manage dry eye symptoms, but long-term drug exposure may cause serious sight threatening side effects including cataract and glaucoma^1^. By contrast, epigallocatechin gallate (EGCG) is a natural compound obtained from green tea extract that may help maintain a healthy metabolism^2^. Because of its bioactivities, EGCG has been investigated as an anti-inflammatory and antioxidant agent in human corneal epithelial cell culture model^3^ and murine dry eye model^4^. These earlier observations indicate that EGCG is a potent candidate for therapeutic applications in topical ocular pharmacological treatment of DED.

Currently, eye drop instillation in patients with various ocular disorders including dry eye remains the primary method in clinical ophthalmology. Results of a clinical trial show that 4 weeks of treatment with trehalose eye drops six times daily protects human corneal epithelial cells from death of desiccation^5^. In another study, both vitamin A eye drops and topical cyclosporine A 0.05% treatments twice daily for 3 months are found to significantly improve vision in patients with dry eye syndrome compared to the control group receiving preservative-free artificial tears alone^6^. In addition, topical 0.03% tacrolimus eye drops twice a day (every 12 h) in the lower conjunctival sac can successfully enhance tear stability in dry eye patients^7^. Although eye drop instillation is the most widely preferred way to dispense ophthalmic medication, low precorneal residence and ocular bioavailability of topically administered drugs are the main challenges with this type of dosage form. It has been documented that the rapid renewal rate of lachrymal fluid (1–3 μl/min) together with the blinking reflex, leads to short residence time of drugs in the precorneal space (<1 min)^8^. In order to avoid the unnecessary toxicity and economic burden associated with high dosage frequency, it is highly desirable to develop suitable drug delivery systems that prolong the residence time of bioactive molecules at the site of action.

Over the past few years, our group has been working on the development of biodegradable *in situ* gelling gelatin-*g*-poly(*N*-isopropylacrylamide) (GN) carriers for intracameral delivery of antiglaucoma medications^9–12^. In a rabbit model of experimental glaucoma, intraocular administration of pilocarpine using GN can more effectively improve ocular bioavailability than intracameral free drug injection. While no therapeutic benefit is noted for free drug-treated groups at 2 weeks postoperatively, extended pharmacological responses (i.e., reduction of intraocular pressure and pupil size and preservation of corneal endothelial cell morphology) are seen in animals receiving drug-containing injectable polymer depot. Due to the viscosity building effects of gelatin^13^, the grafting of thermo-responsive polymer segments onto proteinaceous networks results in excellent adherence of the GN carriers, thereby implying their potential applications as *in situ* forming delivery systems on ocular surface. Based on these considerations, we hypothesize that biodegradable *in situ* gelling GN materials will also enhance pharmacological efficacy of medication in the management of dry eye symptoms. To the best of our knowledge, the development of ophthalmic drop of EGCG-loaded GN for the treatment of dry eye syndrome is yet to be explored. Hence, the purpose of this work was to evaluate the possible contribution of biodegradable *in situ* gelling carriers to the improved therapeutic effect of EGCG after topical ocular administration in dry eye animals. The EGCG-loaded GN samples were verified by chemical and phase transition characterizations and *in vitro* degradation and drug release studies. Biocompatibility tests and anti-inflammatory and antioxidant activity studies were also performed with human corneal epithelial cell cultures. A rabbit model of experimental dry eye induced by benzalkonium chloride (BAC) was used to examine ocular drug bioavailability in relation to disease progression (Figure S1).

## Results

### Characterization studies

**Figure 1:**
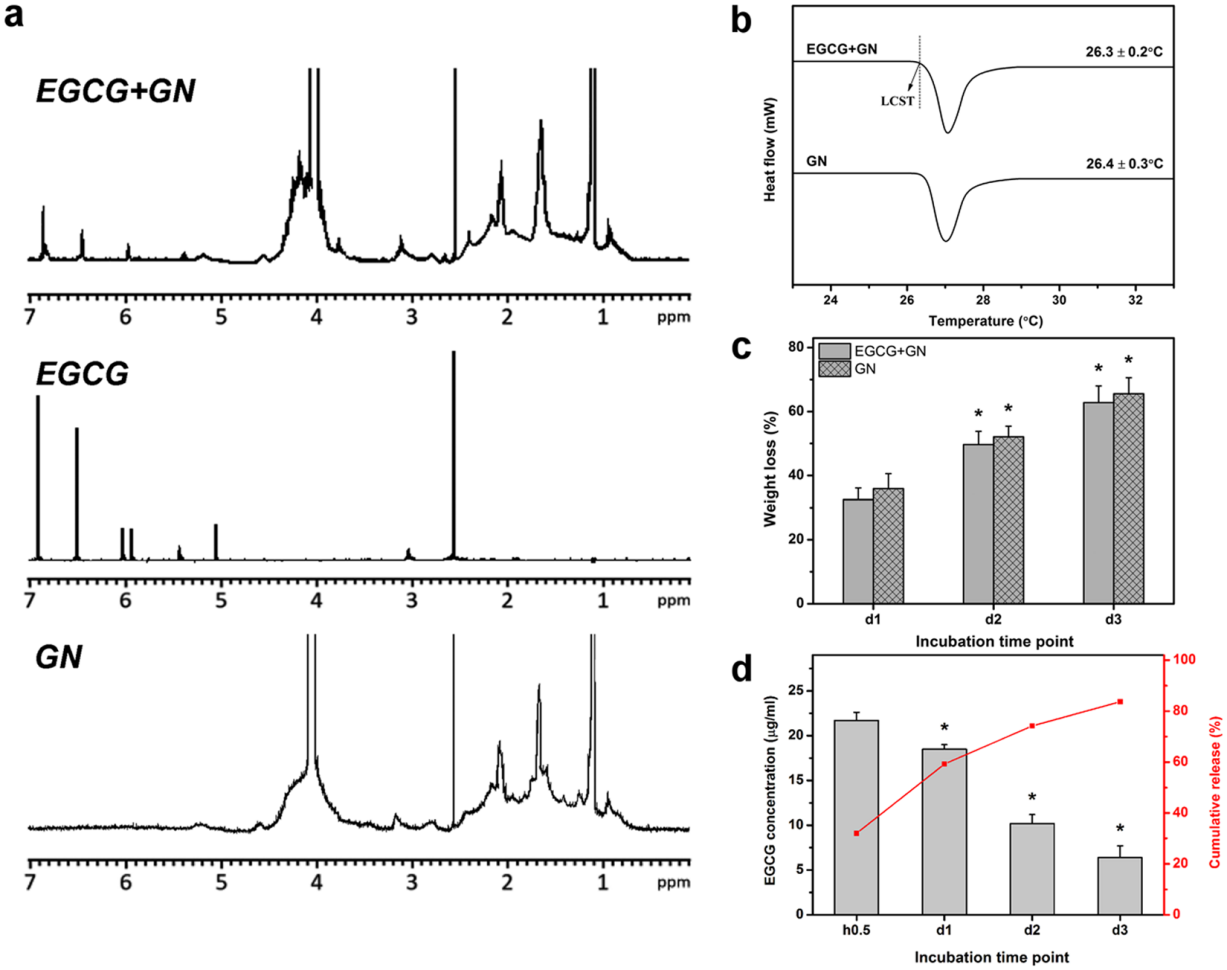
Characterization of EGCG-loaded GN. (**a**) ^1^H NMR spectra of GN, EGCG, and EGCG + GN samples in DMSO. (**b**) DSC thermograms of GN and EGCG + GN samples in ATS. Each LCST data point represents the average of four different values. (**c**) Time-course of weight loss of GN and EGCG + GN samples after incubation at 32 °C in ATS containing MMP-9. An asterisk indicates statistically significant differences (*P < 0.05; *n* = 5) for the mean value of weight loss compared to the value at the previous time point. Incubation time point: day (d). (**d**) The concentration of EGCG and cumulative release percentage from GN carriers at 32 °C in ATS containing MMP-9. An asterisk indicates statistically significant differences (*P < 0.05; *n* = 4) for the mean value of released EGCG concentration compared to the value at the previous time point. Incubation time point: hour (h); day (d).

To characterize EGCG-loaded GN samples, Fourier transform infrared (FTIR) spectroscopy was performed. In our previous study^9^, FTIR spectrum of GN copolymers has been recorded to identify the functional group. As shown in Figure S2, the materials of GN groups revealed several characteristics bands at 3278 cm^−1^ (N-H stretching), 2986 cm^−1^ (C-H stretching), 1634 cm^−1^ (amide I, C=O stretching), 1546 cm^−1^ (amide II, N-H bending), 1459 and 1373 cm^−1^ (symmetric and antisymmetric deformation of -C(CH_3_)_2_), and 1235 cm^−1^ (amide III, N-H bending), which are typtical of those observed for biodegradable backbone of gelatin networks and thermo-responsive PNIPAAm segments. In addition, the EGCG molecules revealed several absorption bands at 1528 cm^−1^ (C=C aromatic ring vibration), 1346 cm^−1^ (O-H in-plane bending vibration), and 1146 cm^−1^ (O-H aromatic ring vibration)^14^. The spectra of EGCG + GN samples displayed the characteristic peaks of amides, isopropyl group, and aromatic compound with many hydroxyl groups. In addition, no new peaks were observed in the spectrum, indicating the absence of chemical interaction between the drug and carrier material^15^. Hydrogen-1 nuclear magnetic resonance (^1^H NMR) spectroscopy was further used to confirm the drug-loading process. The chemical shift scale was referenced against internal DMSO-d6 at 2.6 ppm. As shown in Fig. 1a, a list of chemical shift assignments for the GN was given as follows: 1.1, 1.7, and 2.1 ppm for different protons of NIPAAm component^16^. Major spectral peaks occurring at 2.9–3.1, 5.1, 5.9–6.1, 6.5, and 6.9 ppm were assigned to C-ring CH_2_, C-ring CH, A-ring CH, B-ring CH, and D-ring CH, respectively, which are typical of those found in the EGCG^17^. Our data demonstrated successful preparation of EGCG-loaded GN. Figure 1b shows the results of phase transition experiments in the drug delivery system dissolved in artificial tear solution (ATS). The lower critical solution temperature (LCST) represents the temperature at which a polymer chain shows a coil-to-globule transition in aqueous solution^18^. No significant difference was found in the LCST between GN (26.4 ± 0.3 °C) and EGCG + GN (26.3 ± 0.2 °C) groups (*P* > 0.05). On the other hand, irrespective of presence or absence of EGCG, the GN carrier materials demonstrated continued degradation as seen from the gradual increase in weight loss over time (*P* < 0.05) (Fig. 1c). At each time point, the extent of degradation from GN and EGCG + GN groups revealed that drug loading does not lead to any significant changes in the weight loss of samples incubated in ATS containing matrix metalloproteinase-9 (MMP-9) (*P* > 0.05). Our results also showed that the polymeric carriers may contribute to the stability of EGCG^19^.

After temperature triggered drug encapsulation, the amount of EGCG loaded into carrier materials was determined to be 67.9 ± 1.8 μg. In addition, Fig. 1d shows the release profile of EGCG from GN carrier material in ATS containing enzyme. The drug concentration at 0.5, 24, 48, and 72 h was 21.7 ± 0.9, 18.5 ± 0.5, 10.2 ± 1.0, and 6.4 ± 1.3 μg/ml, respectively, indicating initial burst and subsequent continuous release profiles. The cumulative release of EGCG from biodegradable *in situ* gel forming formulation was 83.7% of the original encapsulated amount after 3 days.

### *In vitro* biocompatibility studies

**Figure 2:**
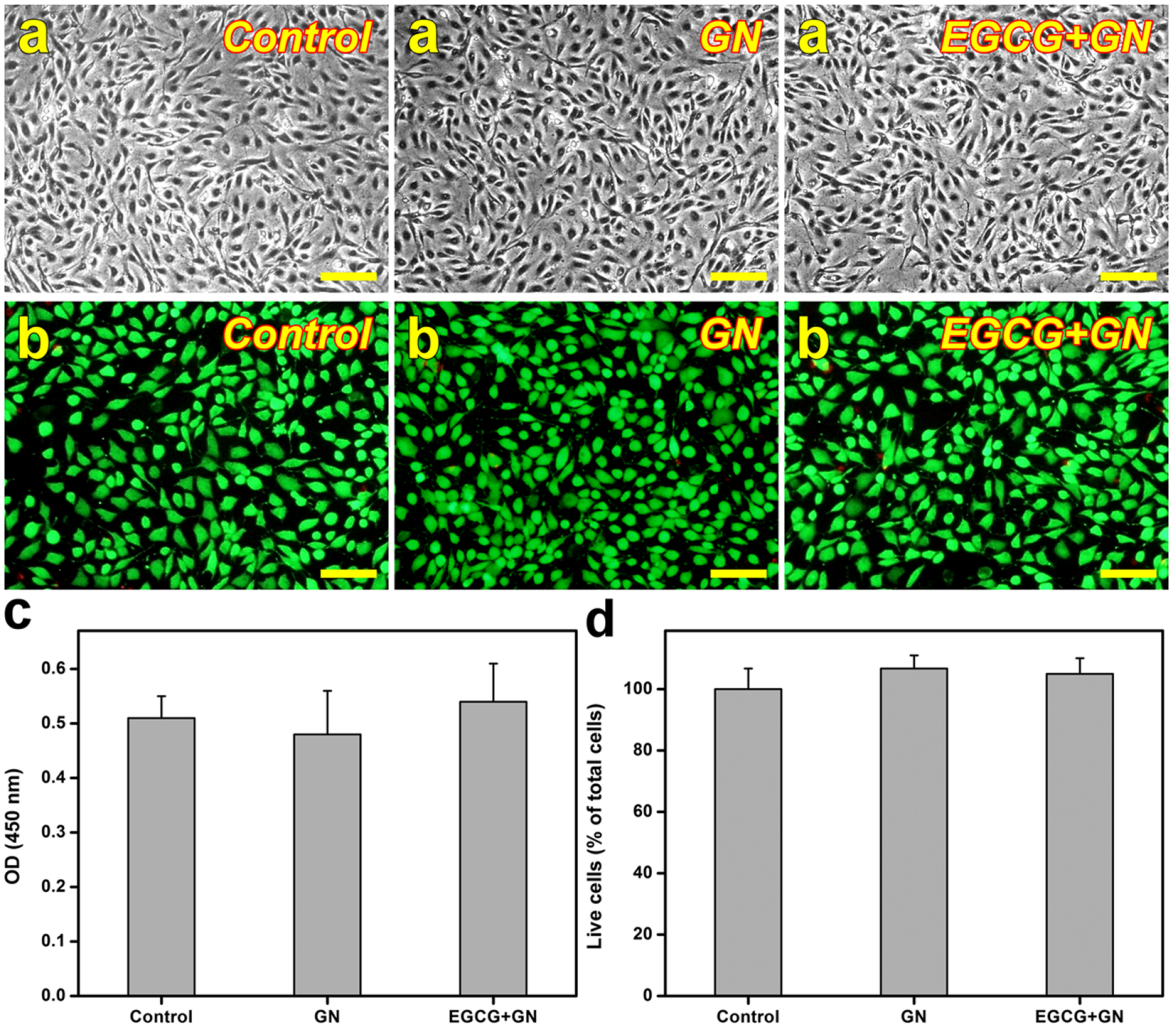
*In vitro* biocompatibility studies. (**a**) Phase-contrast micrographs of HCE-2 cell cultures. The pattern of cell growth in controls (without test samples) after a 3-day exposure to GN and EGCG + GN samples. Scale bars: 100 μm. (**b**) Viability of HCE-2 cell cultures was determined by staining with Live/Dead Viability/Cytotoxicity Kit in which the live cells fluoresce green and dead cells fluoresce red. Fluorescence images of cells in controls (without test samples) after 3 days of exposure to GN and EGCG + GN samples. Scale bars: 100 μm. (**c**) The OD value at 450 nm for HCE-2 cells exposed to GN and EGCG + GN samples for 3 days. Control: without test samples. Values are mean ± standard deviation (*n* = 4). (**d**) Mean percentage of live cells in the HCE-2 cultures as measured by live/dead assay after a 3-day exposure to GN and EGCG + GN samples. Control: without test samples. Values are mean ± standard deviation (*n* = 3).

It is highly desirable to determine the potential toxicity of drug-loaded polymeric carriers before their use in the treatment of DED. Figure 2a shows representative images of HCE-2 cell cultures photographed after 3 days of incubation. In the control, GN, and EGCG + GN groups, the cells appeared healthy and exhibited typical epithelial morphology. As shown in Fig. 2b, prominent green fluorescence was noted in control cultures, indicating a large percentage of live cells. The staining patterns of cells exposed to the GN and EGCG + GN samples could not be distinguished from the control groups. There were no significant differences between the control, GN, and EGCG + GN groups on proliferative capacity (Fig. 2c) and cell viability (Fig. 2d) (P > 0.05). The quantitative results are consistent with the qualitative observations.

### Anti-inflammatory and antioxidant activity studies

**Figure 3:**
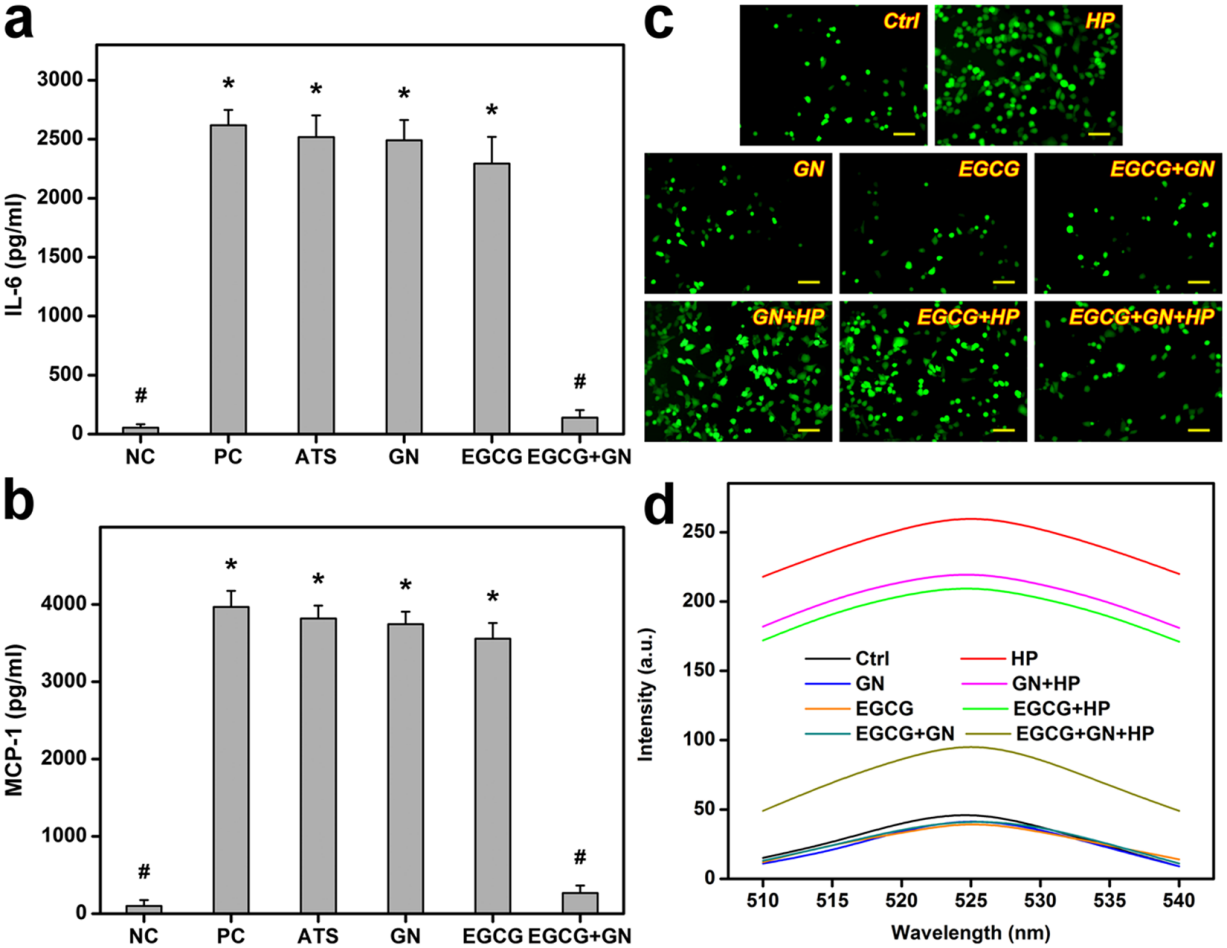
Anti-inflammatory and antioxidant activity studies. Level of (**a**) IL-6 and (**b**) MCP-1 released from HCE-2 cultures after incubation with ATS, GN, EGCG, and EGCG + GN for 3 days. Unstimulated and IL-1β-stimulated cells without contacting the test samples were the negative controls (NC) and positive controls (PC). Values are mean ± standard deviation (*n* = 4). *P < 0.05 vs NC and EGCG + GN groups; ^#^ *P* < 0.05 vs PC, ATS, GN, and EGCG groups. (**c**) Fluorescent images of the HCE-2 cells after incubation with GN, EGCG and EGCG + GN samples for 24 h and further exposure to H_2_O_2_ for 24 h. The cells exposed to 0 (Ctrl group) or 100 (HP group) μM H_2_O_2_ for 24 h following 24 h of incubation in the absence of the test samples were used for comparison. Scale bars: 50 μm. (**d**) Intracellular levels of ROS were measured by the fluorescence intensity of DCFH-DA, with a microplate reader. Quantification results were the mean of four independent experiments.

Among various flavonoids, EGCG is known to be one of the most powerful nutritional molecules that exhibit anti-inflammatory and antioxidant effects^5^. Here, the expressions of pro-inflammatory cytokines such as interleukin-6 (IL-6) (Fig. 3a) and monocyte chemotactic protein-1 (MCP-1) (Fig. 3b) were determined by enzyme-linked immunosorbent assay (ELISA). The measured concentration of IL-6 in the unstimulated (NC group) and IL-1β-stimulated (PC group) HCE-2 cells without contacting any samples was 54.3 ± 30.2 and 2617.8 ± 129.5 pg/ml, respectively. Furthermore, in the NC groups, the detected MCP-1 level was 101.0 ± 75.1 pg/ml, which was significantly lower than that of the PC (3966.4 ± 207.2 pg/ml) groups (*P* < 0.05). Our data demonstrate successful IL-1β-induced corneal epithelial cell inflammation. After 3 days of treatment of the HCE-2 cells with ATS alone or EGCG solution, the persistency of high pro-inflammatory cytokine levels was noted. While the GN carriers did not down-regulate IL-6 and MCP-1 expressions in IL-1β-stimulated cultures (*P* > 0.05), the EGCG-containing GN samples significantly reduce the expression levels of secreted pro-inflammatory cytokines (*P* < 0.05). In this work, the radical scavenging ability of drug-loaded polymer samples was investigated using DPPH method according to decolorization of stable radical in the presence of antioxidants (Figure S4). The GN carriers exhibited 20.4 ± 1.7% inhibition of the DPPH radical, mainly because of the ability of cysteine in gelatin amino components to donate protons^18^. In the EGCG + GN groups, the percentage radical inhibition was 81.9 ± 2.6%, which was significantly higher than that of the GN groups (*P* < 0.05). Figure 3c shows the results of intracellular reactive oxygen species (ROS) production induced by hydrogen peroxide stimulation. The GN, EGCG, and EGCG + GN groups displayed similar fluorescence patterns to Ctrl groups, indicating good cytocompatibility of test samples. By contrast, the HP (i.e., 24 h of exposure of cells to 100 μM hydrogen peroxide following 24 h of incubation in the absence of test sample) groups demonstrated more prominent green fluorescence as a result of the ROS accumulation triggered by H_2_O_2_ exposure. In this *in vitro* oxidative stress model, the cells of EGCG + GN + HP groups emitted less fluorescence than those of GN + HP and EGCG + HP groups. As shown in Fig. 3d, the order of increasing intracellular ROS level measured by spectrofluorometer was the following: HP > GN + HP > EGCG + HP > EGCG + GN + HP > Ctrl. The quantitative data of fluorescence intensity support qualitative observations.

### Clinical observations

**Figure 4:**
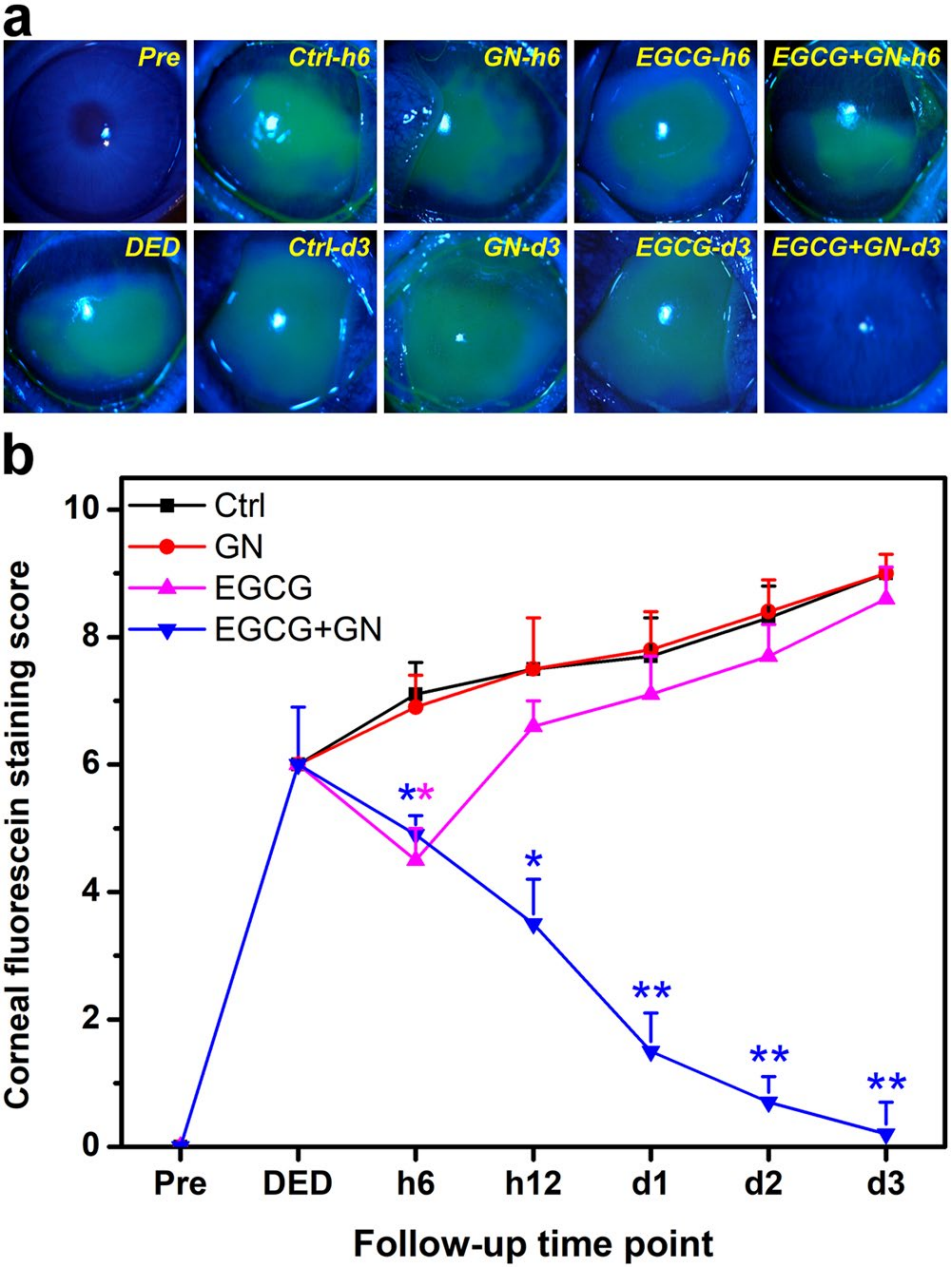
Corneal fluorescein staining measurements. Corneal fluorescein staining (**a**) images and (**b**) scores of rabbit eyes at preoperation (Pre) and after dry eye (DED) induction, and those with experimentally induced DED after topical administration of GN, EGCG, and EGCG + GN solutions. Dry eye animals receiving ATS without polymer and drug serve as control groups (Ctrl). Asterisks indicate statistically significant differences (*P < 0.05; **P < 0.005; *n* = 6) as compared with the Ctrl groups. Follow-up time point: hour (h); day (d).

**Figure 5:**
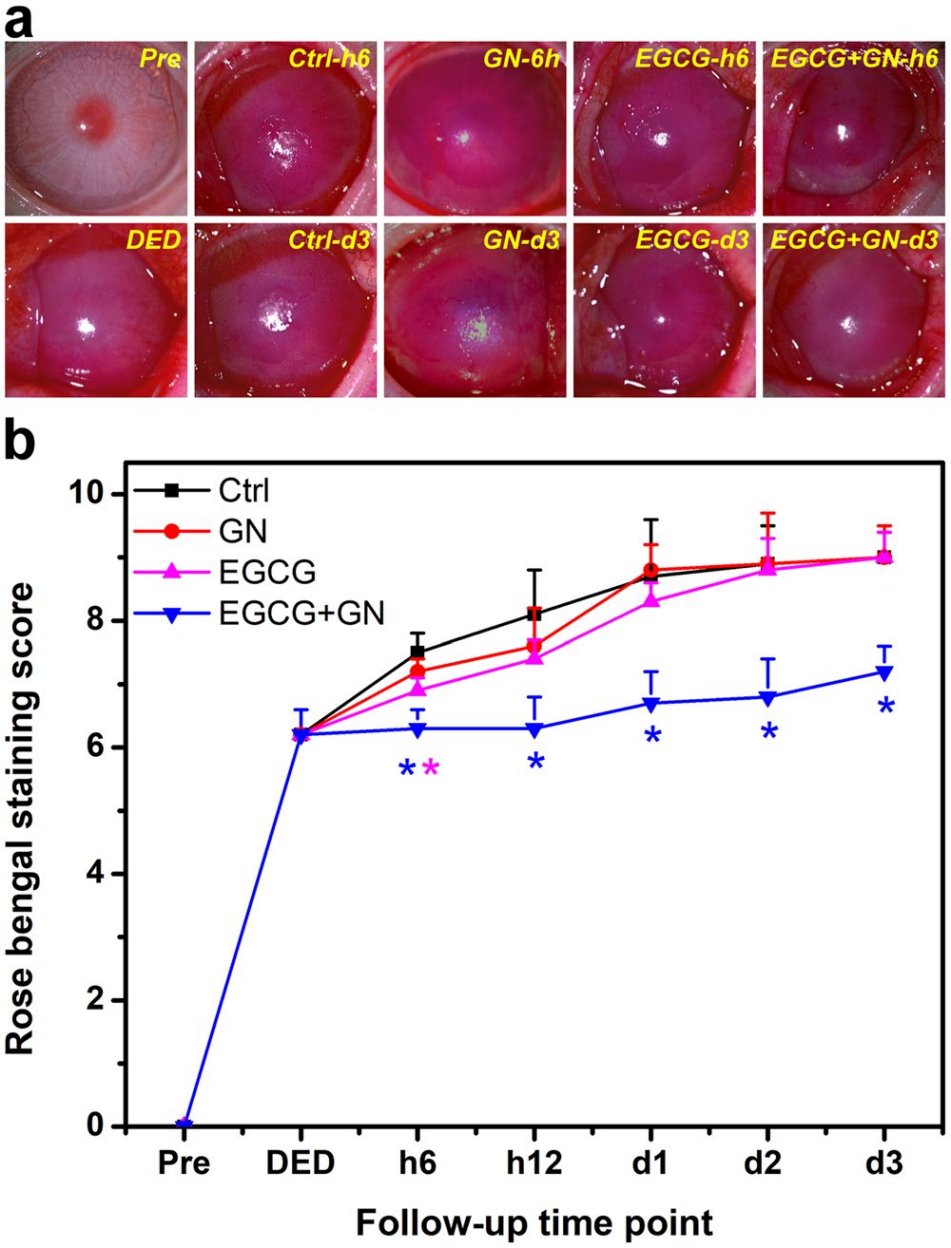
Rose bengal staining measurements. Rose bengal staining (**a**) images and (**b**) scores of rabbit eyes at preoperation (Pre) and after dry eye (DED) induction, and those with experimentally induced DED after topical administration of GN, EGCG, and EGCG + GN solutions. Dry eye animals receiving ATS without polymer and drug serve as control groups (Ctrl). Asterisks indicate statistically significant differences (*P < 0.05; *n* = 6) as compared with the Ctrl groups. Follow-up time point: hour (h); day (d).

**Figure 6:**
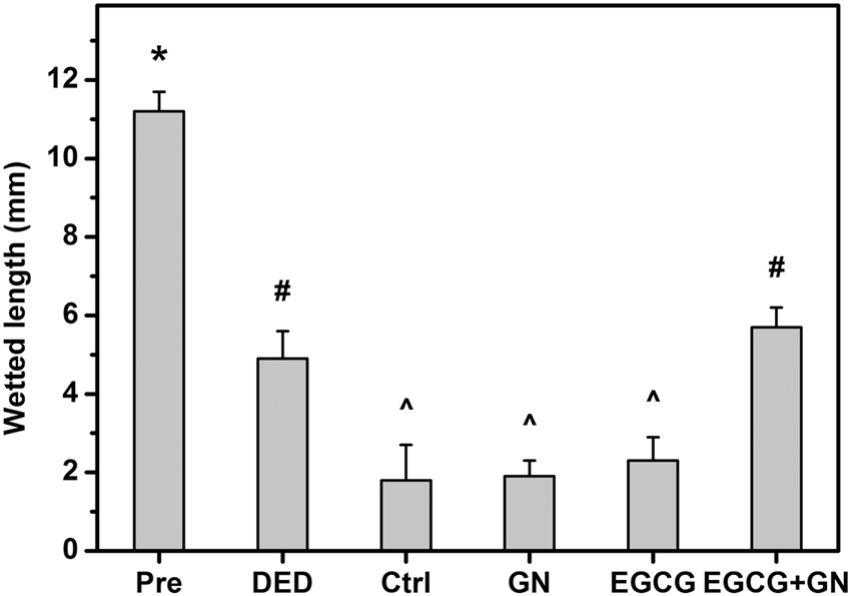
Schirmer tests. The wetted length of the Schirmer paper strip for the rabbit eyes at preoperation (Pre) and those with experimentally induced dry eye (DED) 3 days after topical administration of GN, EGCG, and EGCG + GN solutions. Dry eye animals receiving ATS without polymer and drug serve as control groups (Ctrl). Values are mean ± standard deviation (*n* = 6). *P < 0.05 vs all groups; ^#^ *P* < 0.05 vs Pre, Ctrl, GN, and EGCG groups; ^^^ *P* < 0.05 vs Pre, DED, and EGCG + GN groups.

In clinical ophthalmology, fluorescein eye staining test is a useful tool in evaluating corneal epithelial defects of patients with DED since it can provide important information regarding the level of tissue damage^20^. Figure 4 shows the results of corneal fluorescein staining studies. At preoperation, there was no uptake of fluorescein stain by the cornea of Pre groups. By contrast, in the DED groups, the corneal epithelial defect was present with positive fluorescein staining after experimental dry eye induction (Fig. 4a). During the follow-up, intense positive fluorescent signals could still be detected in the Ctrl (topical instillation of ATS alone) and GN groups. However, for both EGCG and EGCG + GN groups, the fluorescein-stained area of the cornea was reduced at 6 h postoperatively. It is interesting to note that with increasing time from 6 h after ocular administration, most of the fluorescein appeared to be decreasing in rabbits receiving EGCG-containing GN while significant fluorescein retention was visualized in corneal tissues treated with EGCG solutions. The results of quantitative analysis of corneal fluorescein staining are shown in Fig. 4b. After 14 days of desiccation treatment, a relatively high staining score was achieved compared to the preoperative status. Similar levels of fluorescein staining were observed between the Ctrl, GN and EGCG groups at each follow-up time point except for 6 h. The score in the EGCG + GN group was significantly lower than that of the Ctrl group over the course of pharmacological treatment (*P* < 0.05). Furthermore, rose bengal staining of the ocular surface is able to demonstrate the loss of cytoprotection by an intact mucin layer. As shown in Fig. 5a, rose bengal could not stain the healthy ocular surface at preoperation. In the DED groups, positive rose bengal staining was observed due to the absence of normal preocular tear film. During the follow-up, moderate to severe staining was noted in Ctrl, GN and EGCG groups. However, several hours after ocular administration of EGCG-containing GN, there were marked improvements in rose bengal staining patterns from dry eye rabbits. Figure 5b shows the results of quantitative analysis of rose bengal staining. In the DED groups, significant increases in staining score were also found compared to the preoperative values (*P* < 0.05). The occurrence of ocular surface penetration of rose bengal dye in Ctrl animals led to further discomfort, which was evidenced by apparent addition of a certain amount of score. Within postoperative 12 h to 3 days, the rose bengal score between the Ctrl, GN, and EGCG groups did not show a significant difference (*P* > 0.05), and the value was maintained at a high level. By contrast, significant decreases in staining score were observed for the animals receiving EGCG-containing GN (*P* < 0.05). The Schirmer score is another important quantitative measure of aqueous tear production. Figure 6 shows the wetted length of the Schirmer paper strip after being placed in the cul-de-sac for 3 min. At preoperation, the wetted length was 11.2 ± 0.5 mm. It was significantly higher than all the other groups treated with BAC (*P* < 0.05). As such, the mean wetted length was 1.8 ± 0.9 mm for the Ctrl animals and that for the GN and EGCG-treated rabbits was 1.9 ± 0.3 and 2.3 ± 0.6 mm, respectively, indicating no significant difference in tear secretion between these three groups at 3 days postoperatively (*P* > 0.05). By contrast, significant increases in wetted length were found for the dry eye animals receiving EGCG-containing GN (*P* < 0.05). The EGCG + GN group had a comparable Schirmer score to that of rabbits immediately following BAC treatment, suggesting that drug delivery by topical application of biodegradable *in situ* gelling copolymers can effectively prevent further tear evaporation during the follow-up.

### Histological examinations

**Figure 7:**
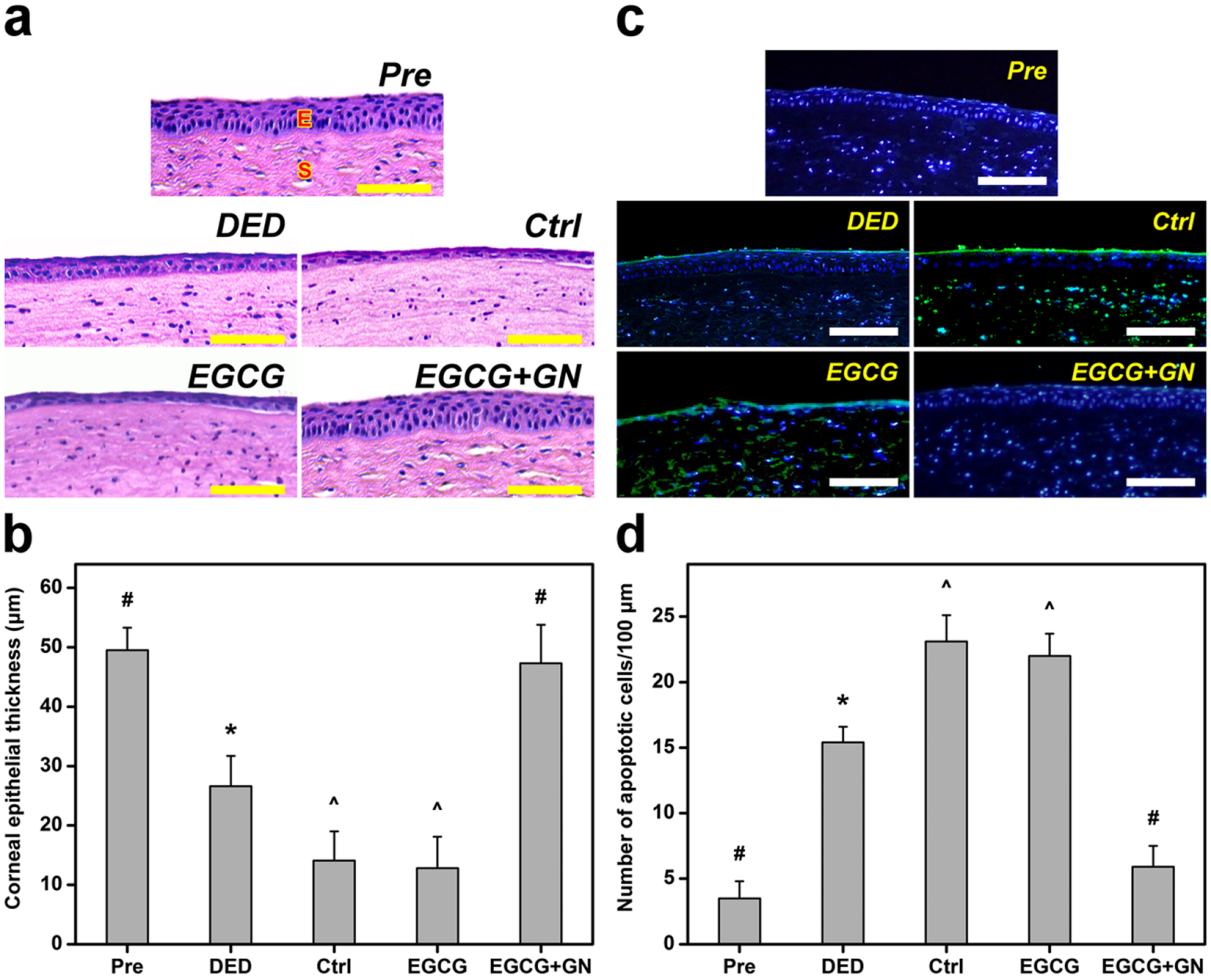
H&E and TUNEL assays. (**a**) Histological images, (**b**) thickness values, (**c**) fluorescence images, and (**d**) apoptotic cell numbers of corneal epithelium in rabbit eyes at preoperation (Pre) and those with experimentally induced dry eye (DED) 3 days after topical administration of EGCG and EGCG + GN solutions. Dry eye animals receiving ATS without polymer and drug serve as control groups (Ctrl). Sections are stained with (**a,b**) H&E and (**c,d**) TUNEL and DAPI. Scale bars: 100 μm. (**a**) E: epithelium; S: stroma. (**c**) Blue fluorescence is DAPI nuclei staining. Green fluorescence is TUNEL-positive nuclei staining. (**b,d**) Values are mean ± standard deviation (*n* = 6). *P < 0.05 vs all groups; ^#^ *P* < 0.05 vs DED, Ctrl, and EGCG groups; ^^^ *P* < 0.05 vs Pre, DED, and EGCG + GN groups.

**Figure 8:**
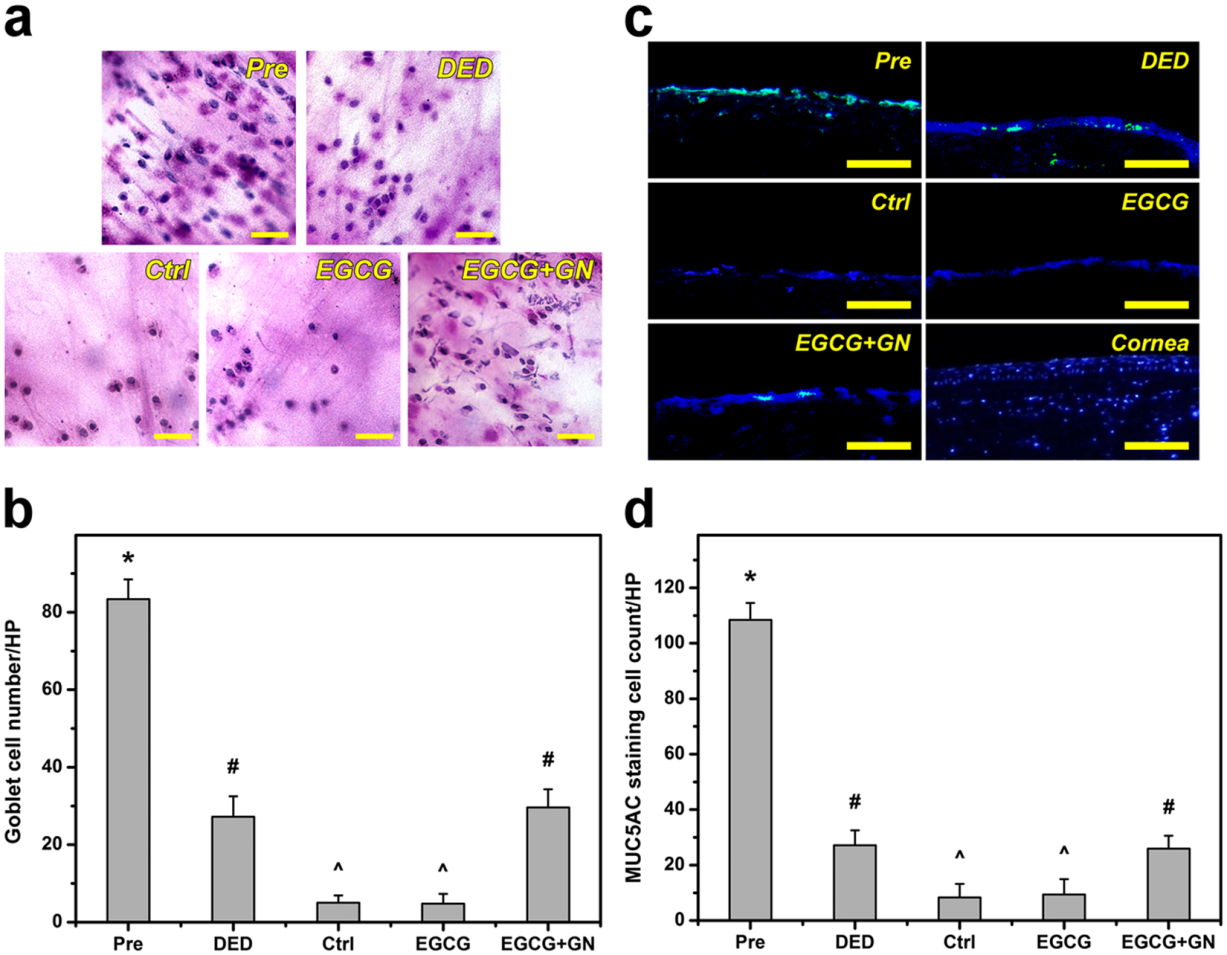
Impression cytology and immunofluorescence for MUC5AC. (**a**) Conjunctival impression cytological images, (**b**) goblet cell numbers, (**c**) immunofluorescence images, and (**d**) MUC5AC staining cell counts in rabbit eyes at preoperation (Pre) and those with experimentally induced dry eye (DED) 3 days after topical administration of EGCG and EGCG + GN solutions. Dry eye animals receiving ATS without polymer and drug serve as control groups (Ctrl). Sections are stained with (**a,b**) hematoxylin (blue) and PAS (pink) and (**c,d**) MUC5AC (green) and DAPI (blue). Scale bars: (**a**) 40 μm; (**c**) 100 μm. (**c**) In the Cornea groups, no MUC5AC-positive cells are detected and used as a negative staining control. (**b,d**) Values are mean ± standard deviation (*n* = 6). *P < 0.05 vs all groups; ^#^ *P* < 0.05 vs Pre, Ctrl, and EGCG groups; ^^^ *P* < 0.05 vs Pre, DED, and EGCG + GN groups.

In order to examine the tissue structural alterations in response to topically administered EGCG-containing GN, the corneal epithelial thickness of dry eye rabbits was measured. As shown in Fig. 7a, the cornea of Pre groups was multilayered^21^. However, in the DED groups, the corneal epithelium exhibited abnormal architecture. At 3 days postoperatively, the rabbits receiving ATS alone (Ctrl groups) or EGCG solution (EGCG groups) showed thinner corneal epithelial layers than animals after experimental dry eye induction. The corneal epithelial tissue integrity was similar in the DED and EGCG + GN groups. Figure 7b shows the results of corneal epithelial thickness determined from H&E staining images. In the Pre groups, the thickness was 49.5 ± 3.8 μm, which is in accordance with the reported value for normal rabbit corneal epithelium^22^. After BAC treatment, the animal eyes had a significantly reduced thickness (*P* < 0.05). The tissue thickness in DED (26.6 ± 5.1 μm) groups was significantly higher than that of Ctrl (14.1 ± 4.9 μm) and EGCG (12.8 ± 5.3 μm) groups (*P* < 0.05), indicating further increase in epithelial cell loss. There were no significant differences between the Pre and EGCG + GN groups on corneal epithelial thickness (*P* > 0.05). Although the exact pathogenesis mechanism of DED is not yet completely clear, the increased tear osmolarity may possibly contribute to the production and release of inflammatory mediators from ocular surface epithelial cells into the tear film, thereby leading to cellular apoptosis^23^. In this study, corneal tissue sections were also evaluated for apoptosis using a terminal deoxynucleotidyl transferase (TdT)-mediated dUTP nick end labeling (TUNEL) assay. Fluorescence microscopic images of all studied groups demonstrated cell nuclei stained with 4′,6-diamidino-2-phenylindole (DAPI) in blue (Fig. 7c). While only a few TUNEL-labeled apoptotic cells with fragmented DNA (green) were visualized in the Pre and EGCG + GN groups, prominent green fluorescent signals could be detected in the DED, Ctrl, and EGCG groups. The number of TUNEL-positive corneal epithelial cells is shown in Fig. 7d. Experimental induction of DED could trigger a large amount of apoptotic cells in rabbit corneal epithelium, which corresponds with those found in other animal studies using mice^24^ or rats^25^. In both Ctrl and EGCG groups, significant increases in the number of TUNEL-positive cells were found compared to the values of the DED groups (*P* < 0.05). However, the proportion of apoptotic cells showed no significant difference between the Pre and EGCG + GN groups (*P* > 0.05). It has been documented the etiology of corneal epithelial alteration and ocular surface tissue damage in dry eye patients that involve accumulation of oxidative stress^26^. Results of corneal epithelial histological studies indicate good anti-inflammatory response and antioxidant potential for the EGCG released from GN carriers at 3 days of follow-up. On the other hand, periodic acid-Schiff (PAS) staining of impression cytology specimens was performed to explore conjunctival goblet cell density in dry eye rabbits treated with topically administered medications. As shown in Fig. 8a, the goblet cells could be stained pink by PAS dye and the nuclei of surrounding epithelial cells would be stained blue by hematoxylin. In the Pre groups, abundant goblet cells were oval and had intensely PAS-positive cytoplasm^27^. By contrast, goblet cells were markedly decreased as demonstrated by conjunctival impression cytological imaging of BAC-treated rabbits. In particular, PAS-positive cells were barely recognized in both Ctrl and EGCG groups. For the rabbits receiving ATS alone or EGCG solution, the relatively low cell numbers were obtained at 3 days postoperatively compared to the preoperative status (*P* < 0.05) (Fig. 8b). A similar level of goblet cells was present between the DED and EGCG + GN groups (*P* > 0.05). The quantitative results are consistent with the qualitative observations. Immunofluorescence staining was also used to detect the difference in the expression of MUC5AC (i.e., the most abundant ocular surface mucin). As shown in Fig. 8c, the cells of conjunctiva stained by MUC5AC antibody were clearly visible in the Pre groups. For both DED and EGCG + GN groups, fewer MUC5AC-positive cells could be identified. In the Ctrl animals and those treated with EGCG solutions, the MUC5AC staining markedly declined. The negative staining control showed absence of MUC5AC expression in normal corneal tissue. The order of increasing MUC5AC staining cell count was the following: Pre > DED > Ctrl (Fig. 8d). In the EGCG groups, there was no increase in the number of MUC5AC-positive cells as compared to that from Ctrl eyes (*P* > 0.05). Interestingly, the MUC5AC expression in the EGCG + GN groups could be restored to an extent corresponding to the amount of MUC5AC-positive cells in the animals of DED groups (*P* > 0.05). Given that poor tear film stability is associated with reduction in goblet cell density and mucin deficiency on the ocular surface^23^, our results of conjunctival impression cytology and immunofluorescence staining for MUC5AC (Fig. 8) validate the findings of rose bengal staining (Fig. 5) and Schirmer test (Fig. 6).

## Discussion

The therapeutic action of epigallocatechin gallate (EGCG) is linked to its strong bioactivities (antioxidant and anti-inflammation capacity), which may be helpful in treating preservative (i.e., benzalkonium chloride)-induced rabbit dry eye condition clinically similar to that in human. The copolymers of gelatin and PNIPAAm have been used as *in situ* gellable artificial extracellular matrices for cultivation of smooth muscle cells^28^ and mesenchymal stem cells^29^. In our laboratory, the thermo-responsive and biodegradable features of injectable GN depots are beneficial to the design of new antiglaucoma formulations based on pilocarpine^10^. To improve pharmacological bioavailability, the ophthalmic drop of EGCG-loaded GN is being researched in this work for its effectiveness in the treatment of dry eye syndrome. Given that understanding the temperature triggered sol-gel phase transition and enzymatic degradation behaviors of GN materials may make important contributions to the control of EGCG delivery performance, it is highly desirable to investigate the LCST and weight loss of carriers after their chemical characterization by FTIR and NMR. Investigators have examined the influence of drug incorporation on the thermal properties of carrier materials using differential scanning calorimetry (DSC) technique and found that the thermograms for both naringenin-loaded solid lipid nanoparticles and blank nanocarriers are similar due to the dominant role of solid lipid core material in endothermic reactions^30^. The present findings are compatible with this earlier report. Furthermore, MMP-9 is known to be the primary matrix-degrading enzyme (i.e., gelatinase) produced by the corneal epithelium. A previous study has demonstrated the presence of a high level of MMP-9 (~2000 ng/ml) in tear fluid of the patients with DED^31^. Therefore, *in vitro* degradation tests are conducted at physiological temperature^32^, with MMP-9 concentration corresponding to the level in dry eye tears. Since the PNIPAAm is not biodegradable in nature^12^, the data regarding the continued weight loss possibly reflect the biodegradability of gelatin components in the GN materials under the action of proteinases. As reported in the literature, there is no significant difference in matrix degradation between the lidocaine-loaded and drug-free bioerodible polymer groups^33^. Our findings are in accordance with their study and suggest that the EGCG resides within the GN carrier does not perturb the interaction between the matrix and enzyme. The functionality of GN materials has been demonstrated to give excellent results in the development of biodegradable *in situ* gelling delivery system for the intracameral pilocarpine administration^9^. By means of temperature triggered gel formation and enzymatic degradation of carrier matrix, the antiglaucoma medication can be effectively encapsulated into and released from the delivery vehicle. Here, biodegradable *in situ* gel forming formulation based on EGCG is tested by pharmacological studies. The drug encapsulation efficiency is almost 100% using *in situ* gelling carriers since the EGCG is theoretically present inside the GN after temperature triggered gel formation^16^. The initial burst release of encapsulated EGCG in first 30 min is noted. Since no degradation of GN materials occurs within this period, desorption of drug localized on the surface of delivery carriers may be responsible for the observed behavior. The present findings also suggest that fast temperature triggered capture of EGCG and progressive gelatin degradation of carrier allow high drug payload and cumulative release.

The drug-containing delivery systems are designed to be placed onto the ocular surface. Therefore, the corneal epithelial cellular responses to these foreign substances are examined. Results of WST-1 and live/dead assays demonstrate good compatibility of GN carriers, which support our earlier observations that the GN copolymers are well tolerated by the corneal endothelial cell cultures^11^. In addition, the EGCG compound released from biodegradable *in situ* gel forming formulation does not decrease the viability of HCE-2 cell cultures, suggesting no cytotoxicity due to drug exposure. Since the molecular weight of EGCG compound is 458 g/mol, the drug concentrations at different time points within 3 days of release are calculated to be between 14.0 and 47.4 μM. As reported in the literature, no significant change in viability level of renal tubular epithelial cells treated with EGCG at 12.5–50 μM^34^. The present findings also indicate that below the threshold concentration, the EGCG molecules can be considered as non-irritants. On the other hand, given that the occurrence of oxidative and/or inflammatory reactions of the ocular surface in dry eye patients is frequently related to disease progression^35^, the *in vitro* therapeutic potential of EGCG-containing GN is studied. Our results show that the drug-loaded polymeric carriers have nearly four times greater DPPH radical scavenging capacity than their counterparts without EGCG. On the other hand, the inflammatory and oxidative effects cannot be suppressed by administration of EGCG solutions to the cell cultures. One possible explanation is that under many cell culture conditions, the half-life of EGCG is relatively short (about 2 h), leading to insufficient therapeutic concentration^36^. In contrast, strong anti-inflammatory and antioxidant activities of EGCG released from GN hydrogels are demonstrated using *in vitro* models of inflammation and oxidative stress in corneal epithelial cells, implying that the polymeric carriers are beneficial to protect the drug molecules from destabilization^19^. Investigators have previously shown that green tea polyphenol EGCG at 3–30 μM significantly inhibits pro-inflammatory cytokine release in corneal epithelial cells^3^. Furthermore, 1 μM EGCG is found to be sufficient for effective scavenging 50% of the DPPH radical^37^. It has been reported that pretreatment with 5–50 μM EGCG certainly attenuates nicotine-induced ROS activation in endothelial cells^38^. According to the data of drug release profiles, EGCG concentration at each study time point can suppress the secretion of pro-inflammatory cytokines and production of oxidative stress molecules by stimulated corneal epithelial cells.

There are currently a variety of clinically available diagnostic techniques for assessing dry eye patients such as fluorescein and rose bengal staining and Schirmer tests. As reported in the literature, the patients who afflicted with the symptoms of dry eye are usually featured by ocular surface abnormalities (fluorescein vital staining score of ≥3 or rose bengal staining score of ≥3) and tear film abnormalities (Schirmer test value of ≤5 mm)^39^. In this study, a dry eye animal model is successfully established by topical ocular administration of 0.1% BAC (i.e., quaternary ammonium compound) twice daily for 14 days. Pathogenesis of this experimental model of DED is attributed to that BAC can hasten the drying of preocular tear film and induce dry eye syndrome in rabbits with signs of corneal and conjunctival damage and tear secretion deficiency^27^. Poor tear film stability is also found to be highly correlated with reduction in goblet cell density and mucin deficiency on the ocular surface. Results of clinical observations and histological examinations show that the Ctrl animals receiving topical instillation of ATS alone may have continued disease progression during the follow-up. In addition, only GN treatment is not significant effective for the management of dry eye symptoms compared to Ctrl group. After ocular administration of EGCG solutions for more than 6 h, the gradual rise in corneal fluorescence and rose bengal staining scores is noted. In particular, abnormal preocular tear film and deficient tear and mucin production are also found with increasing follow-up duration. It has been documented that topical ophthalmic drug delivery remains challenging due to the dynamic barriers (i.e., tear dilution, lymphatic clearance, and conjunctival blood flow)^15^. One possible explanation for our observations is that the eye drop dosage form is commonly restricted by the very short precorneal residence time, resulting in limited pharmacological response. By contrast, for the rabbits bearing EGCG-containing GN, sustained drug release ameliorates BAC-induced corneal epithelial defects by suppressing cellular inflammation, stress, and apoptosis. At 3 days postoperatively, the EGCG molecules are able to effectively prevent further tear evaporation and loss of mucin-secreting goblet cells in dry eye animals. Although topical ocular application of drug using biodegradable *in situ* gelling carriers has been shown to achieve longer therapeutic action, the EGCG-containing GN cannot completely relieve dry eye symptoms, as indicated by a relatively high rose bengal staining score and comparable goblet cell number and ocular surface mucin expression to those of rabbits immediately following BAC treatment. Our findings possibly reflect the differences in cellular responses to drug treatment. In contrast to conjunctival goblet cells, corneal epithelial cells have a rapid regenerative capacity due to high renewal ability of resident stem cells^40^. Therefore, the corneal epithelial defects can be treated through cell-mediated tissue reconstruction accompanied by anti-inflammatory and antioxidant effects of EGCG released from GN hydrogels. The future interest will be towards the investigation of topical ocular delivery of EGCG combined with other medications that can promote goblet cell regeneration. For the first time, here, we demonstrate that single EGCG drop administration using biodegradable *in situ* gelling carriers can significantly improve pharmacological response in dry eye rabbits in comparison to instillation of free drug.

## Conclusions

In summary, we show that biodegradable *in situ* gelling carrier has a pivotal role in the design of EGCG-based formulation for the treatment of dry eye syndrome. Loading of EGCG into GN hydrogels does not affect the phase transition temperature, biodegradability, and biocompatibility of copolymer carriers for topical ocular drug delivery. After release from GN hydrogels, the EGCG compounds can exhibit anti-inflammatory and antioxidant effects, thereby offering therapeutic benefits for the treatment of DED. Results of clinical observations and histological examinations demonstrate that limited disease progression associated with cellular inflammation and oxidative stress is seen in rabbits receiving EGCG-loaded GN compared with the animals treated by topical instillation of artificial tear drops or administration of free drug solutions at 3 days postoperatively. The findings of present study suggest that GN copolymer is responsible for the enhanced pharmacological efficacy of EGCG in a rabbit dry eye model and shows great potential as topical ocular drug delivery system with extended release characteristics. The information about the effect of single EGCG drop administration using biodegradable *in situ* gelling carrier on dry eye relief presents an opportunity for further development of pharmacological interventions.

## Methods

### Materials

Type A gelatin (300 Bloom), Epigallocatechin gallate (EGCG), 2,2′-diphenyl-1-picrylhydrazyl (DPPH), hydrogen peroxide, matrix metalloproteinase-9 (MMP-9, EC 3.4.24.35), and benzalkonium chloride (BAC) were purchased from Sigma-Aldrich (St. Louis, MO, USA). *N*-isopropylacrylamide (NIPAAm), from Acros Organics (Geel, Belgium), was purified by recrystallization from n-hexane. FNC Coating Mix (i.e., a fibronectin/collagen mixture) was obtained from Athena ES (Baltimore, MD, USA). Keratinocyte serum-free medium (KSFM) was purchased from Gibco-BRL (Grand Island, NY, USA). All the other chemicals were of reagent grade and used as received without further purification.

### Characterization of EGCG-loaded GN

The gelatin-*g*-PNIPAAm (GN) copolymers were synthesized by using carbodiimide coupling chemistry to attach carboxylic end-capped PNIPAAm onto the aminated gelatin^9^. According to the protocols described in our previous publication^11^, the feed molar ratio of NH_2_ groups in the aminated gelatin to COOH groups in the carboxylic end-capped PNIPAAm was controlled at 0.36 for preparation of GN samples. The reaction was allowed to proceed at 25 °C for 24 h. Then, the reaction product was precipitated at 50 °C, followed by centrifugation and resuspension in deionized water. To remove unreacted components, the suspension was exhaustively dialyzed (MWCO 50000, Spectra/Por^®^ Dialysis Membrane, Rancho Dominguez, CA, USA) against deionized water at 4 °C for 4 days. The GN graft copolymer was lyophilized at −50 °C and kept in a closed vessel at room temperature. For temperature triggered drug encapsulation, 630 μl of GN solutions (10% w/v) were prepared by dissolving the solutes in artificial tear solution (ATS) containing 124 mM Na^+^, 133 mM Cl^−^, 24 mM HCO_3_^−^, 30 mM K^+^, 0.7 mM Mg^2+^, 0.7 mM Ca^2+^, 0.35 mM glucose, 4.5 mM urea, 3.5 mM lactate, and 0.2 mM pyruvate^41^, mixed with 70 μl of EGCG solutions (0.1% w/v) at 20 °C. The drug-incorporated hydrogels were formed by heating the solutions at 32 °C (i.e., the ocular surface temperature in dry eye patients)^32^.

The Fourier transform infrared (FTIR) spectroscopy of various samples was performed using a FT-730 ATR-FTIR Spectrophotometer (Horiba, Japan). The spectra were recorded between 3700 and 850 cm^−1^ with a resolution of 8 cm^−1^. Hydrogen-1 nuclear magnetic resonance (^1^H NMR) spectra were recorded for various samples dissolved in DMSO-d6 using a Bruker Avance DRX 500 NMR instrument (Taipei Medical University, Taipei, Taiwan, ROC). The ^1^H chemical shift scale was referenced against internal DMSO-d6 at 2.6 ppm. The phase transition temperatures of various samples were examined using a differential scanning calorimeter (DSC) (TA Instruments, New Castle, DE, USA). Programmed heating was carried out at 3 °C/min in the temperature range of 20 °C–40 °C under a nitrogen gas flow. Lower critical solution temperature (LCST) was determined as the onset point of the endothermic peak. Results were averaged on four independent runs. For degradation measurements, the test samples were dried to constant weight (*W*_i_) in vacuo and immersed in ATS containing 2000 ng/ml of MMP-9 (physiological level present in dry eye tear) at 32 °C. At specific time intervals, the degraded samples were collected and further dried in vacuo to measure the weight (*W*_d_). The percentage of weight loss (%) was calculated as ((*W*_i_ − *W*_d_)/*W*_i_) × 100. Results were the average of five independent measurements. For the evaluation of drug encapsulation levels, the EGCG-loaded GN samples were transferred to an empty vial at 20 °C. The redissolved samples were analyzed by high performance liquid chromatography (HPLC) using a L-2400 UV detector and L-2130 pump (Hitachi, Tokyo, Japan) and a Mightysil RP-18 column (Kanto Chemical, Tokyo, Japan). The eluant peak was detected by measuring absorbance at 280 nm^42^. To determine the amount of entrapped drug in each sample, photometric reading was referenced to a standard curve of peak area versus EGCG concentration (0.1–300 μg/ml). Results were averaged on four independent runs. Stability of EGCG was also determined by reverse-phase HPLC as previously described^43^. Drug release studies were performed similarly as the degradation tests earlier using 2000 ng/ml of MMP-9 at 32 °C. Release buffer was collected at predetermined time points and analyzed by HPLC. EGCG concentrations, after being released from GN carriers, were calculated using a calibration curve. Results were the average of four independent measurements. The cumulative release percentage of drug at each time point was determined by dividing the amount of the averaged released EGCG by the total amount of the loaded EGCG and multiplied by 100.

### *In vitro* biocompatibility studies

In this study, HCE-2 cells, a human corneal epithelial cell line (ATCC No. CRL-11135), were purchased from the American Type Culture Collection (Manassas, VA, USA). The cells were seeded on tissue culture plastics precoated with FNC Coating Mix, and maintained in regular growth medium containing KSFM, 0.05 mg/ml bovine pituitary extract, 5 ng/ml epidermal growth factor, 500 ng/ml hydrocortisone, and 0.005 mg/ml insulin^44^. Cultures were incubated in a humidified atmosphere of 5% CO_2_ at 37 °C. The medium was changed twice a week. Cells were subcultured by trypsinization at a split ratio of 1:3. The HCE-2 cells with a density of 5 × 10^4^ cells/well were seeded into 24-well plates by 1 ml/well. Using cell culture inserts (Falcon 3095, Becton Dickinson Labware, Franklin Lakes, NJ, USA), each well of a 24-well plate was divided into two compartments. The GN and EGCG-loaded GN samples prepared by temperature triggered *in situ* gel method as aforementioned were respectively added to the inner well of the double-chamber system containing 2000 ng/ml of MMP-9 at 37 °C to examine the cultures after 3 days of exposure to test samples. The cells in regular growth medium without any samples served as control groups.

Cell morphology was observed by phase-contrast microscopy (Nikon, Melville, NY, USA). In addition, the cell cultures were examined using a Live/Dead Viability/Cytotoxicity Kit (Molecular Probes, Eugene, OR, USA). The living cells were identified through signals of green fluorescence from the intracellular esterase activities due to cleavage of calcein AM. By contrast, a red fluorescence was produced via binding of EthD-1 to the nucleic acids in dead cells with damaged cell membranes. Cell proliferation was determined by using WST-1 assay (Roche Diagnostics, Indianapolis, IN, USA). After incubation with WST-1 reagent for 4 h at 37 °C, the optical density (OD) value at 450 nm was recorded using a Multiskan Spectrum Microplate Spectrophotometer (ThermoLabsystems, Vantaa, Finland). All experiments were conducted in quadruplicate. For quantification of live/dead cells, the cultures were counted at 100× magnification under fluorescence microscopy (Axiovert 200 M; Carl Zeiss, Oberkochen, Germany). All experiments were performed in triplicate, and the viability of the HCE-2 cell cultures was expressed as the average ratio of live cells to the total number of cells.

### Anti-inflammatory and antioxidant activity studies

HCE-2 cells (5 × 10^4^ cells/well) were seeded in 24-well plates containing regular growth medium and incubated overnight. For interleukin-1β (IL-1β) stimulation, the medium was replaced with the fresh medium containing 1 ng/ml. Using cell culture inserts (Becton Dickinson Labware), each well of a 24-well plate was divided into two compartments. The GN and EGCG-loaded GN samples prepared by temperature triggered *in situ* gel method as aforementioned were respectively added to the inner well of the double-chamber system containing 2000 ng/ml of MMP-9 at 37 °C to examine the IL-1β-stimulated cultures exposed to test samples. Furthermore, the EGCG solutions were administered at 0.1% w/v and were placed in the inner well of a two-chamber culture plate for comparison. In this study, blank experiments (ATS only) were conducted simultaneously to avoid possible interference in absorbance readings. Unstimulated and IL-1β-stimulated HCE-2 cells without contacting any samples also served as the negative control (NC) and positive control (PC) groups, respectively. After 3 days of incubation, the release of interleukin-6 (IL-6) and monocyte chemotactic protein-1 (MCP-1) from cultivated cells into the conditioned medium was detected by the Quantikine enzyme-linked immunosorbent assay (ELISA) kit (R&D Systems, Minneapolis, MN, USA) specific for human IL-6 and MCP-1. Aliquots of the supernatant from each well were collected, and cytokine bioassays were performed according to the manufacturer’s instructions^45^. Photometric readings at 450 nm were measured using the Multiskan Spectrum Microplate Spectrophotometer (ThermoLabsystems). Results were expressed as pg/ml. All experiments were conducted in quadruplicate.

Free radical scavenging activities of GN and EGCG-loaded GN samples were evaluated using DPPH assays^18^. After incubation with DPPH for 30 min, the absorbance of test samples was measured by an UV-Vis spectrophotometer (Thermo Scientific, Waltham, MA, USA) at 517 nm. Calculations of DPPH scavenging activity (%) was based on ((*A*_0_ − *A*_1_)/*A*_0_) × 100, where *A*_0_ is the absorbance of blank DPPH solution at the same reaction conditions in the absence of any samples, and *A*_1_ is the absorbance of DPPH solution in the presence of test samples. All experiments were conducted in quadruplicate. On the other hand, the hydrogen peroxide-induced oxidative stress of HCE-2 cell model was used to measure antioxidant activity. The HCE-2 cells with a density of 5 × 10^4^ cells/well were seeded in 24-well plates and incubated with EGCG solutions, GN and EGCG-loaded GN samples for 24 h in the presence of 2000 ng/ml of MMP-9. Then, the cell cultures were treated with a further incubation of 24 h in medium containing 100 μM hydrogen peroxide. For comparison purpose, the cells were exposed to hydrogen peroxide of 0 μM (Ctrl group) and 100 μM (HP group) for 24 h following 24 h of incubation in the absence of the test samples. Intracellular accumulation of reactive oxygen species (ROS) was measured by oxidative conversion of cell-permeable 2′,7′-dichlorodihydrofluorescein diacetate (DCFH-DA) (Molecular Probes) to fluorescent 2′,7′-dichlorofluorescein (DCF)^46^. The HCE-2 cells in the culture wells were incubated with 10 μM DCFH-DA solutions at 37 °C for 1 h. The DCF fluorescence imaging (Ex. 488 nm; Em. 525 nm) was acquired with a fluorescence microscope (Carl Zeiss). Furthermore, the fluorescence reading was done with a multi-mode microplate reader (BioTek Instruments, Winooski, VT, USA) to detect the difference in the fluorescence intensity. All experiments were conducted in quadruplicate.

### Animal studies

All animal procedures were approved by the Institutional Review Board of Chang Gung University (IACUC approval number: CGU16-059) and were conducted in accordance with the ARVO Statement for the Use of Animals in Ophthalmic and Vision Research. Thirty adult New Zealand white rabbits (National Laboratory Animal Breeding and Research Center, Taipei, Taiwan, ROC), weighing 3.0–3.5 kg and 16–20 weeks of age, were used for this study. Animals were healthy and free of clinically observable ocular surface disease. Surgical operation was performed in the single eye of animals, with the normal fellow eye. Experimental dry eye model was induced by topical administration of 0.1% BAC twice daily for 14 days^27^. For comparison, 6 dry eye rabbits in DED groups were included in the design of the study. In the three test groups (GN, EGCG and EGCG + GN) of animals (6 rabbits/group), the dry eye rabbits received topical instillation of 50 μl of GN (10% w/v) solutions, 50 μl of EGCG (0.1% w/v) solutions, and 50 μl of a mixture containing EGCG (0.1% w/v) and GN (10% w/v) solutions, respectively. Without treatment with any polymers and drugs, the remaining 6 rabbits with experimental DED served as a control group (Ctrl). To determine the therapeutic efficacy during the follow-up of 3 days, the rabbits were anesthetized intramuscularly with 2.5 mg/kg body weight of tiletamine hydrochloride/zolazepam hydrochloride mixture (Zoletil; Virbac, Carros, France) and 1 mg/kg body weight of xylazine hydrochloride (Rompun; Bayer, Leverkusen, Germany).

Clinical observations were performed by corneal fluorescein and rose bengal staining. The ocular surface was examined and graded by slit-lamp biomicroscopy (Topcon Optical, Tokyo, Japan) following topical application of 2 μl of 1% fluorescein sodium or rose bengal into the conjunctival sac. For the animals with corneal fluorescein staining under cobalt blue light on slit-lamp evaluation, the fluorescence intensity was recorded on the cornea. In addition, the degree of rose bengal staining in the temporal and nasal conjunctiva and cornea was quantified. A standardized 4-point scale (0 = none, 1 = mild, 2 = moderate, and 3 = severe) was applied in each of three areas to analyze the results^39^. Total staining scores were in the range of 0–9. On the other hand, the tear production was assessed by using Schirmer test. Each Schirmer tear test strip (Color Bar Schirmer Tear Test, EagleVision, Memphis, TN, USA) was inserted into the external third of the lower eyelid of the eye without topical anesthesia. After 3 min, the wetted length in millimeters of the strip was measured and taken as the test score^47^.

Animals were euthanized with CO_2_ gas at the end of experiments (i.e., 3 days). The excised rabbit corneas were processed for histological examinations^48^. The samples were fixed in 4% paraformaldehyde in phosphate-buffered saline, dehydrated in a graded series of ethanol solutions, embedded in paraffin, and cut into 5 μm sections. Thin sections were stained with hematoxylin and eosin (H&E) and examined under light microscope (Carl Zeiss) to evaluate the corneal epithelial thickness. In addition, to evaluate apoptosis of corneal epithelial cells *in vivo*, tissue sections were analyzed by terminal deoxynucleotidyl transferase (TdT)-mediated dUTP nick end labeling (TUNEL) assay (Roche Diagnostics)^49^. After fixation with 4% paraformaldehyde, the specimens were permeabilized in 0.1% Triton X-100 in 0.1% sodium citrate for 2 min on ice and incubated with a mixture of TdT solution and fluorescein isothiocyanate dUTP solution in a humidified chamber for 1 h at 37 °C. The negative controls were incubated with distilled water in place of TdT enzyme. To visualize cell nuclei, sections were counterstained with 4′,6-diamidino-2-phenylindole (DAPI; Vector, Peterborough, England) and observed under fluorescence microscope (Carl Zeiss). Three different areas were randomly selected, and the number of TUNEL-positive apoptotic cell nuclei was quantified. Conjunctival impression cytology specimens were also collected by placing circular disks of nitrocellulose filter paper on the nasal and temporal bulbar conjunctiva with the filter paper dull-side down^27^. Then, the samples were stained with hematoxylin and periodic acid-Schiff (PAS) to visualize goblet cells under light microscope (Carl Zeiss). Three different areas were randomly selected, and the number of goblet cells was counted and averaged (cells/high-power [HP] visual field with 400×). On the other hand, immunohistochemical staining of MUC5AC was detected in the tissue sections of the nasal and temporal bulbar conjunctiva^27^. Corneal specimens were also used for comparison. After fixation and blocking with 4% bovine serum albumin, the samples were incubated with a 1:150 dilution of mouse anti-rabbit MUC5AC antibody (Abcam, Cambridge, MA, USA) overnight at 4 °C. The negative controls were incubated without primary antibody. The specimens were washed in phosphate-buffered saline and incubated with fluorescein-conjugated secondary antibody (1:200; Chemicon International, Temecula, CA, USA) for 1 h at room temperature in the dark. Unbound excess labels were removed by rinsing the samples in phosphate-buffered saline. Tissue sections were counterstained with DAPI (Vector) and viewed under fluorescence microscopy (Carl Zeiss). Three different areas were randomly selected, and the number of MUC5AC-positive cells was counted and averaged (cells/high-power [HP] visual field with 400×).

### Statistical analyses

Results were expressed as mean ± standard deviation (SD). Comparative studies of means were performed using a one-way analysis of variance (ANOVA) followed by a Newman-Keuls post hoc test. Significance was accepted with *P* < 0.05.

## Electronic supplementary material

Supplementary Information
