# Supplementary material for: Epigallocatechin Gallate-Loaded Gelatin-g-Poly(N-Isopropylacrylamide) as a New Ophthalmic Pharmaceutical Formulation for Topical Use in the Treatment of Dry Eye Syndrome
Source: Sci Rep. 2017 Aug 24;7:9380. doi: 10.1038/s41598-017-09913-8 (PMC5571197; doi:10.1038/s41598-017-09913-8)
Supplement: Supplementary file 1 — Supplementary Information [file 41598_2017_9913_MOESM1_ESM.pdf]

Supplementary Information

Epigallocatechin Gallate-Loaded Gelatin-*g*-Poly(*N*-Isopropylacrylamide) as a New Ophthalmic Pharmaceutical Formulation for Topical Use in the Treatment of Dry Eye Syndrome

Li-Jyuan Luo[1,†] and Jui-Yang Lai[2,3,4,†,*]

[1]Department of Chemical and Materials Engineering, Chang Gung University, Taoyuan 33302, Taiwan, ROC
[2]Institute of Biochemical and Biomedical Engineering, Chang Gung University, Taoyuan 33302, Taiwan, ROC
[3]Department of Ophthalmology, Chang Gung Memorial Hospital, Taoyuan 33305, Taiwan, ROC
[4]Department of Materials Engineering, Ming Chi University of Technology, New Taipei City 24301, Taiwan, ROC

[*]jylai@mail.cgu.edu.tw
[†]these authors contributed equally to this work

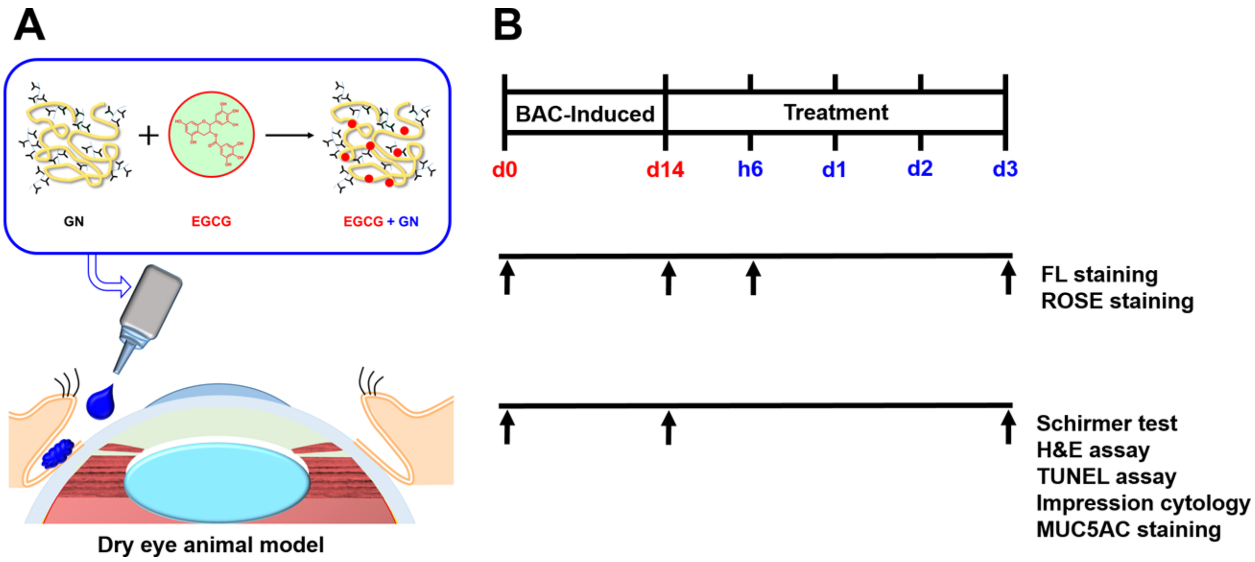

Figure S1. Scheme of experimental design of the study. A BAC-induced rabbit dry eye model was used to examine therapeutic efficacy of short-term topical EGCG-loaded GN for the treatment of dry eye syndrome.

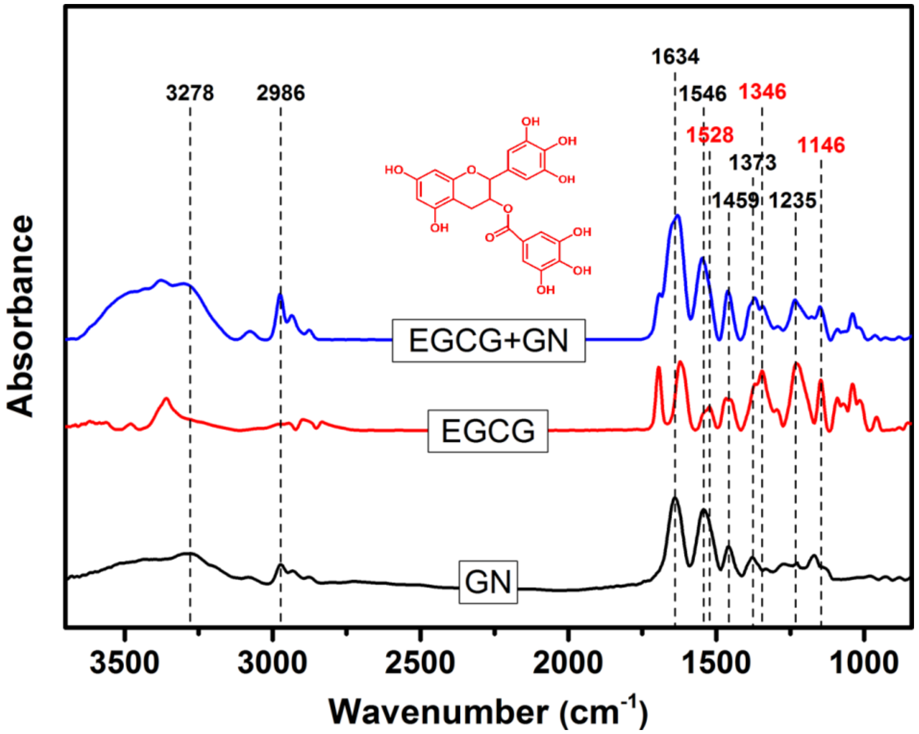

Figure S2. Characterization of EGCG-loaded GN. FTIR spectra of GN, EGCG, and EGCG+GN samples.

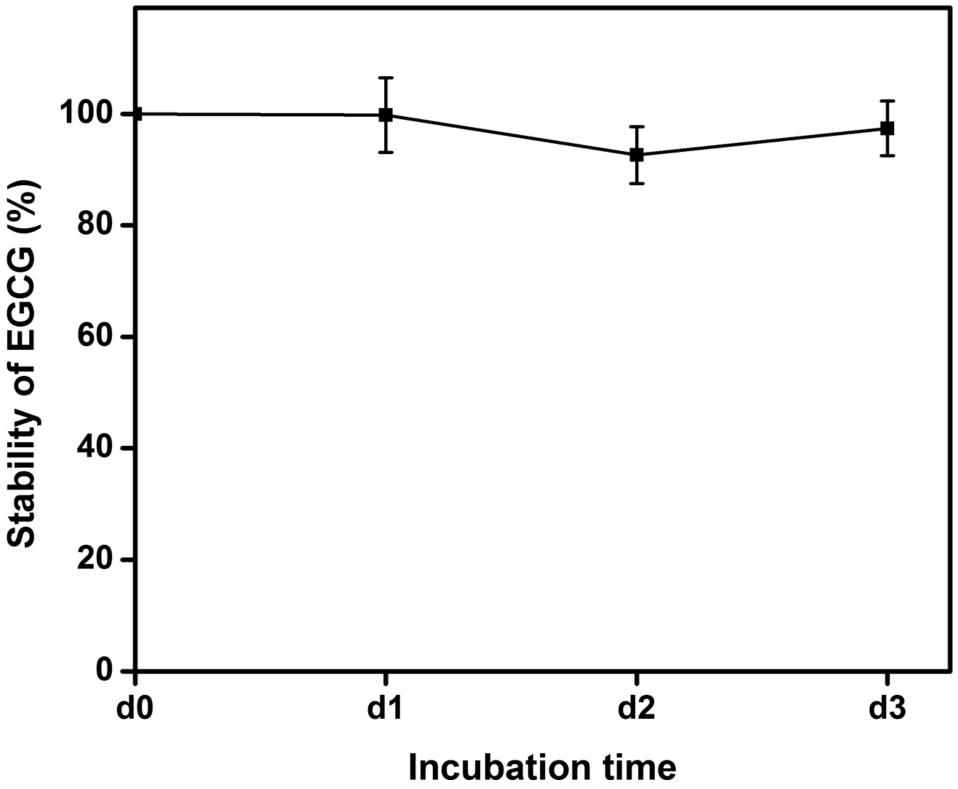

Figure S3. Stability of EGCG encapsulated into GN in ATS with MMP-9. Time-course concentration changes of EGCG was measured by HPLC.

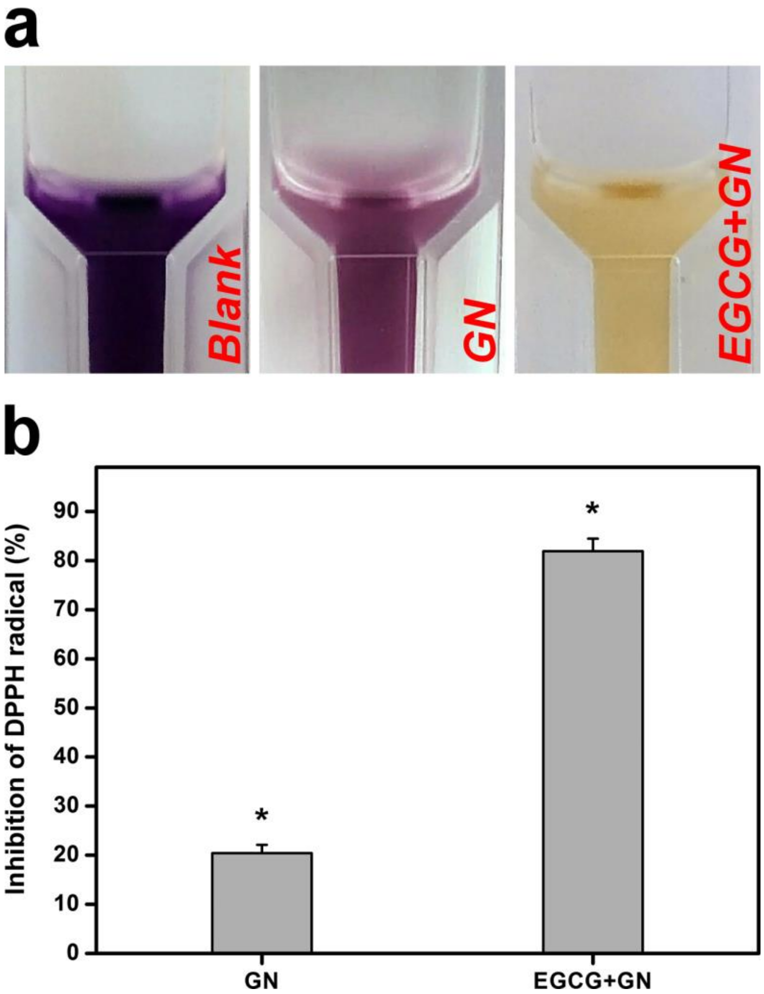

Figure S4. Determination of scavenging activity against DPPH radical. (**a**) Photographs of the reaction of DPPH reagent with GN and EGCG+GN samples. The Blank group is the blank DPPH solution without test samples. (**b**) DPPH scavenging activities of GN and EGCG+GN samples were analyzed by UV-Visible spectrophotometry. Results are expressed as percentage inhibition of the DPPH radical. Values are mean ± standard deviation ($n = 4$). *$P < 0.05$ vs all groups.
